# Apolipoprotein A-I mediates function of follicular regulatory T cells and type 2 follicular helper T in allergic rhinitis

**DOI:** 10.1016/j.waojou.2025.101045

**Published:** 2025-03-24

**Authors:** Xiangqian Qiu, Yinhui Zeng, Jinyuan Li, Qingxiang Zeng, Xi Luo, Wenlong Liu

**Affiliations:** Department of Otolaryngology, Guangzhou Women and Children's Medical Center, Guangzhou Medical University, Guangzhou, 510623, China

**Keywords:** Allergic rhinitis, Follicular regulatory T cells, Type 2 follicular helper T, Apolipoprotein AI

## Abstract

**Background:**

The follicular regulatory T cells (Tfr) and type 2 follicular helper T (Tfh2) play important roles in the pathogenesis of allergic rhinitis (AR). However, its detailed mechanism underlying the regulation of between Tfr and Tfh in AR is unclear. Apolipoprotein AI (Apo-AI), a well-established anti-inflammatory protein, exhibits anti-inflammatory effects on neutrophils, monocytes, macrophages, eosinophils, and type 2 innate lymphoid cells. We sought to investigate the interaction and mechanism between Apo-AI and Tfr/Tfh2 in AR.

**Methods:**

The peripheral Tfh2 and Tfr cells were detected and compared by flow cytometry and their correlation with serum Apo-AI protein expression were analyzed. The effect of Apo-AI on Tfh2 and Tfr cells were determined through detection of functional cytokines and key transcription factors by enzyme-linked immunosorbent assay (ELSIA) or polymerase chain reaction (PCR). A Tfr-Tfh2-B cell coculture system was adopted to investigate the role of Apo-AI. Apo-AI knockout AR mice model was established to verify the results of *in vitro* studies.

**Results:**

The serum Apo-AI concentration was positively correlated with the blood frequencies of Tfr cells and negatively correlated with the blood frequencies of Tfh2 cells in AR patients. Apo-AI inhibited IL-4 and IL-21 protein expression by Tfh2 and promoted IL-10 and TGF-beta protein expression by Tfr. In Tfr-Tfh2-B cell coculture system, Apo-AI attenuated the expression of IL-4, IL-21 and activation-induced cytidine deaminase through inducible costimulator (ICOS)/inducible costimulator ligand (ICOSL) pathways. Apo-AI partially restored the suppressive function of AR-derived Tfr cells. Apo-AI knockout AR mice presented with elevated blood Tfh2 frequencies and decreased blood Tfr frequencies, while administration of anti-ICOSL reversed the effect of Apo-AI.

**Conclusion:**

Apo-AI alleviates AR through the regulation of the function of Tfh2 and Tfr, which may serve as a potential treatment target for AR.

## Introduction

Allergic rhinitis (AR) is caused by a particular inflammatory reaction mediated by immunoglobulin E (IgE) in response to inhaled allergens, which triggers the activation of numerous immune cells.^1^ Recently, a distinctive population of T cells residing within the germinal center (GC) of secondary lymphoid organs and tissues, specifically designated as T follicular helper (Tfh) cells, has been elucidated. These Tfh cells exhibit a pivotal role in the production of interleukin-21 (IL-21) and have emerged as the primary source of interleukin-4 (IL-4) cytokine within secondary lymphoid tissues.^2^ Beyond expressing CXCR5, programmed death-1 (PD-1) has been identified as a characteristic marker of Tfh cells, with implications in modulating the intricate interplay between T and B lymphocytes.^3^

Multiple murine investigations have documented that exposure to allergens fosters the differentiation of T follicular helper (Tfh) cells and stimulates immunoglobulin E (IgE) production.^4^^,^^5^ In human subjects, an aberrant elevation of the Tfh-to-T follicular regulatory (Tfr) cell ratio has been noted in individuals with allergic rhinitis (AR).^6^^,^^7^ Importantly, Tfh cells derived from house dust mite (HDM)-allergic patients display an enhanced capacity to promote IgE synthesis, whereas Tfr cells exhibit diminished immunosuppressive functions.^6^^,^^8^ Subcutaneous immunotherapy (SCIT) intervention led to a notable decrease in IgE production and an augmentation in the ratio of circulating Tfr to Tfh2 cells, suggesting a restoration of regulatory balance and modulation of the allergic response.^6^

Apolipoprotein AI (Apo-AI), the preeminent structural constituent of high-density lipoprotein (HDL) in plasma, exhibits a well-established anti-inflammatory function across diverse inflammatory pathologies, encompassing lupus, Alzheimer's disease, and dermatitis.^9^, ^10^, ^11^ Furthermore, Apo-AI modulates dendritic cell maturation, thereby attenuating T cell activation, indicative of its indirect influence on T cell responses during inflammatory processes.^12^ Gaddis' study posit that elevating Apo-AI levels in humans may favorably regulate the balance between Tfh cell generation and T regulatory (Treg) cell maintenance, ultimately contributing to the mitigation of inflammation, plaque accumulation, and the progression of atherosclerosis.^13^

In this study, we sought to explore the interaction and mechanism between ApoAI and circulating Tfr/Tfh2 in AR through both *in vivo* and *in vitro* studies.

## Methods

### Patient recruitment and symptom evaluation

A total of 30 adult patients with AR exhibiting sensitization to house dust mites (HDM), specifically *Dermatophagoides pteronyssinus* and/or *Dermatophagoides farinae*, and 25 healthy controls were enrolled. The diagnosis of AR adhered to standardized criteria, including a history of characteristic symptoms spanning at least 1 year and confirmation through either a positive skin prick test (SPT) or specific IgE antibodies directed against the aforementioned mite species. The exclusion criteria were rigorously applied, excluding individuals with pregnancy, lactation, immunologic disorders, systemic or topical corticosteroid administration within the preceding 4 weeks, or coexisting allergic conditions such as asthma or atopic eczema.

To quantify the severity of nasal symptoms, a Total Nasal Symptom Score (TNSS) was employed, ranging from 0 (indicating no discomfort) to 3 (signifying extreme discomfort) for each symptom (itching, sneezing, nasal blockage, running nose). Ethical clearance for this study was granted by the local ethics committee (Approval No. 261A01), and informed consent was duly obtained from all participants prior to their enrollment.

### Cell staining

Peripheral blood mononuclear cells (PBMCs) (15 mL) were isolated from AR or control patients or mice utilizing density-gradient centrifugation. The viability of these cells, exceeding 98%, was verified through trypan blue exclusion assay. To mitigate non-specific staining, the PBMCs were pre-incubated with 5% fetal bovine serum for 10 min on ice. Subsequently, a fixable viability dye 700 (BD Bioscience) was applied to exclude dead cells from analysis.

For surface marker staining, the mouse monoclonal antibodies against CD4, PD1, CD25, CXCR3, CCR6, CD3, CD19, CD27, IgD and human recombinant antibody against CXCR5 were incubated with the cells for 30 min at 25 °C. For intracellular Foxp3 staining, cells were fixed with FoxP3 Fix/Perm buffer (BioLegend). Flow cytometric analysis was performed on a BD LSRFortessa™ X-20 instrument (BD Bioscience). CXCR3^−^CCR6^−^CD4^+^CXCR5^+^PD-1^+^ were defined as Tfh2 cells, CD25^+^Foxp3^+^CD4^+^CXCR5^+^PD-1^+^ were defined as Tfr cells.

### Cell sorting

The isolation of naive CD19 + IgD + B cells was accomplished by the depletion of non-naive B cells, utilizing the Naive B Cell Isolation Kit II (Miltenyi Biotec). The purity of CD19 + IgD + naïve B cells was greater than 96%. Tfh2 cells were sorted as CXCR3^−^CCR6^−^CD4^+^CXCR5^+^PD-1^+^ cells and Tfr cells were sorted as CD25^+^Foxp3^+^CD4^+^CXCR5^+^PD-1^+^. Purity of sorted cells was >95%.

### Cell culture

Sorted peripheral Tfh2 cells (5 × 10^4^/well) or Tfr cells (5 × 10^4^/well) were stimulated with different stimulators (1.6 μg/mL Der p1, ProSpec; 10–100 ng/mL Apo-AI, R&D Systems) and supernatant were harvested for further analysis. Moreover, sorted peripheral Tfh2 cells (5 × 10^4^/well) were co-cultured with autologous naïve B cells (2.5 × 10^4^/well) in the presence or absence of autologous or allogenic Tfr cells (5 × 10^4^/well) for a duration of 12 days. This co-culture was conducted in RPMI-1640 medium (Sigma Aldrich, USA), supplemented with 10% heat-inactivated fetal bovine serum (FBS) (Sigma Aldrich, USA) and 1% penicillin/streptomycin (Sigma Aldrich, USA). Additionally, Staphylococcal enterotoxin B (1 μg/mL; Toxin Technology, USA) was included. The cultures were maintained in U-bottomed 96-well plates. In transwell experiments, sorted Tfh cells and autologous naive B cells were seeded in the lower chamber, while autologous Tfr cells were introduced into either the upper or lower transwell chamber as described previously.^14^

In modulating experiments aimed at stimulating or blocking specific pathways, ApoA-I, anti-ICOSL and anti-OX40L (all were 100 ng/mL; R&D Systems) were introduced to the coculture system. Control groups received mouse IgG. Subsequently, culture supernatants were collected for subsequent analyses.

### Quantitative real-time PCR (qRT-PCR) analysis

Total RNA was extracted utilizing the NucleoSpin RNA XS kit (Macherey-Nagel), ensuring the integrity and purity of the RNA template. Subsequently, complementary DNA (cDNA) synthesis was accomplished through reverse transcription, employing Oligo(dT) (12–18mers) primers in conjunction with SuperScript II reverse transcriptase. Quantitative assessment of gene expression was then performed using the ABI PRISM 7300 Detection System, a highly sensitive platform for real-time PCR analysis. The following primer pairs were used: CXCR5: forward, AACGTCCTGGTGCTGGTGA, reverse, CACGGCAAAGGGCAAGA; Bcl6: forward, CCAGCAAAGAAGAAGAGAGACC, reverse, CTGTGGACTAACCAGACCCTTC; Blimp1: forward, AGCTTTCATCCCCTCGTACAAC, reverse, CGCTCAGGCCATTACAATTCAT; STAT5: forward, GAAAGCATG AAAGGGTTGGAG, reverse, AGCAGCAACCAGAGGACTTAC; Foxp3: forward, CAGCACATTCCCAGAGTTCCTC, reverse, GCGTGT GAACCAGTGGTAGATC; CTLA-4: forward, ATG GCTTGCCTTGGATTTCAGCGGCACAAGG, reverse, TCAATTGAT GGGAATAAAATAAGGCTGAAATTGC; activation induced cytidine deaminase (AID): forward, AAAATGTCCGCTGGGCTAAG, reverse, TCGTGGTTTTCTTTGAAGGTCAT; and β-actin: forward, TCCTGTGGCATCCACGAAACT, reverse, GAAGCATTTGCGGTGGACGAT. The amplification protocol comprised an initial denaturation at 95 °C for 2 min, 40 cycles of denaturation at 95 °C for 10 s, annealing for 10 s, and extension at 72 °C for 15 s. Relative gene expression was determined using the 2(-Delta Delta CT) method.

### Enzyme-linked immunosorbent assay (ELISA) for cytokine and immunoglobulin quantification

Commercially available ELISA kits from R&D system (USA) were employed to accurately quantify the concentrations of IgE, as well as cytokines IL-4, IL-21, IL-10, TGF-β, and CXCL13 in the culture supernatants.

### Mouse model and experimental procedures

Female BALB/c or Apo-AI−/− mice (Cyagen, China), aged 8 weeks, were subjected to immunization via intraperitoneal injection with a combination of 100 μg ovalbumin (OVA) and 1.6 mg aluminum hydroxide [Al(OH)_3_] dissolved in phosphate-buffered saline (PBS) on days 0 and 7. Subsequently, an intranasal challenge was administered with 100 μg OVA, with certain mice additionally receiving 10 mg/kg Apo-AI (BioLegend) or 10 mg/kg anti-ICOSL (BioLegend), on days 14, 16, and 19. Following the final challenge, mice were euthanized, peripheral blood, cervical lymph nodes and nasal mucosa were collected for detection of frequencies of Tfh2 and Tfr by flow cytometry as described above and the serum was used for OVA-specific IgE detection.

Nasal turbinate sections of 4–5 μm thickness underwent deparaffinization in xylene and rehydration in graded alcohols. Endogenous peroxidase activity was blocked with 0.3% H_2_O_2_. Antigen retrieval was achieved through autoclave heating in citrate buffer (pH 6.0) for 20 min. Sections were then stained with hematoxylin-eosin. For immunocytochemical analysis, the sections underwent incubation with a polyclonal antibody derived from rabbit, specific for Apo-AI (sourced from Zhongshan Goldenbridge, Beijing, China), diluted at a ratio of 1:200, at 4 °C for an overnight period. Subsequently, on the following day, the specimens were incubated with a secondary antibody, specifically a biotinylated goat anti-mouse/rabbit IgG, which was followed by the application of an avidin-peroxidase conjugate. Following additional rinsing steps, the sections were subjected to staining with 3% diaminobenzidine as the chromogen, counterstained with hematoxylin, and finally mounted with a coverslip.

### Statistical analysis

Statistical analysis was doneby GraphPad Prism 9. The data were presented as means ± standard error mean. Comparisons among groups were finished by Mann-Whitney *U* test, one-way ANOVA, or Student's *t*-test. Correlation analysis was performed using Spearman rank method. A *P*-value less than 0.05 was considered as statistically significant.

## Results

### Serum protein concentration of Apo-AI and its correlation with the frequencies of Tfh2/Tfr in AR

**Fig. 1:**
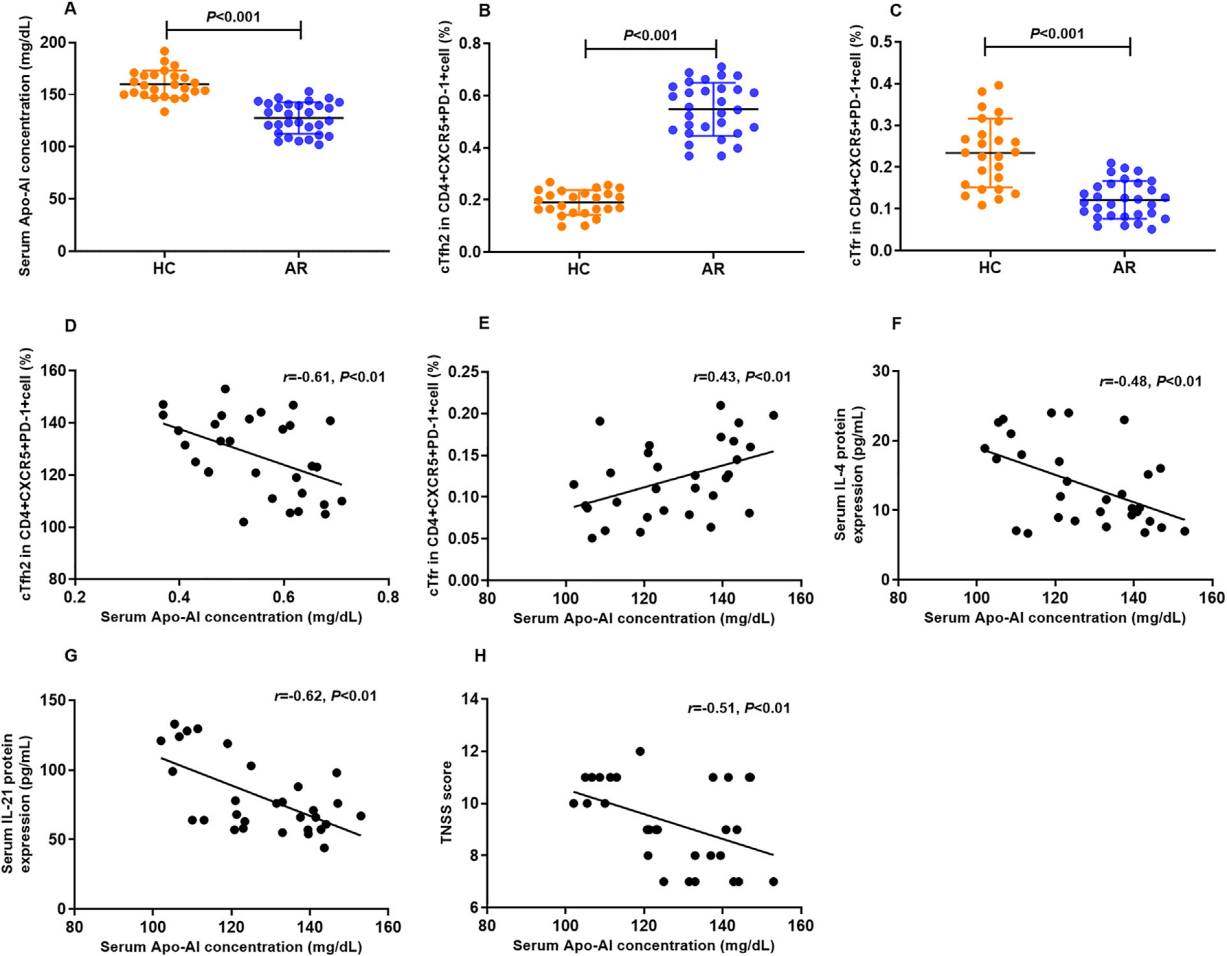
**The serum protein expression of Apo-AI protein in AR and its correlation with the peripheral frequencies of Tfh2 and Tfr cells.** A. The serum protein expression of Apo-AI protein in AR (n = 30) and controls (n = 25) by ELISA. B,C. The peripheral frequencies of Tfh2 and Tfr cells between AR (n = 30) and controls (n = 25) detected by flow cytometry. D-H. The correlation between serum Apo-AI protein and the peripheral frequencies of Tfh2, Tfr, serum IL-4 protein level, serum IL-21 protein level and TNSS score. HC, healthy control, AR, allergic rhinitis, TNSS, total nasal symptom score.

**Fig. 2:**
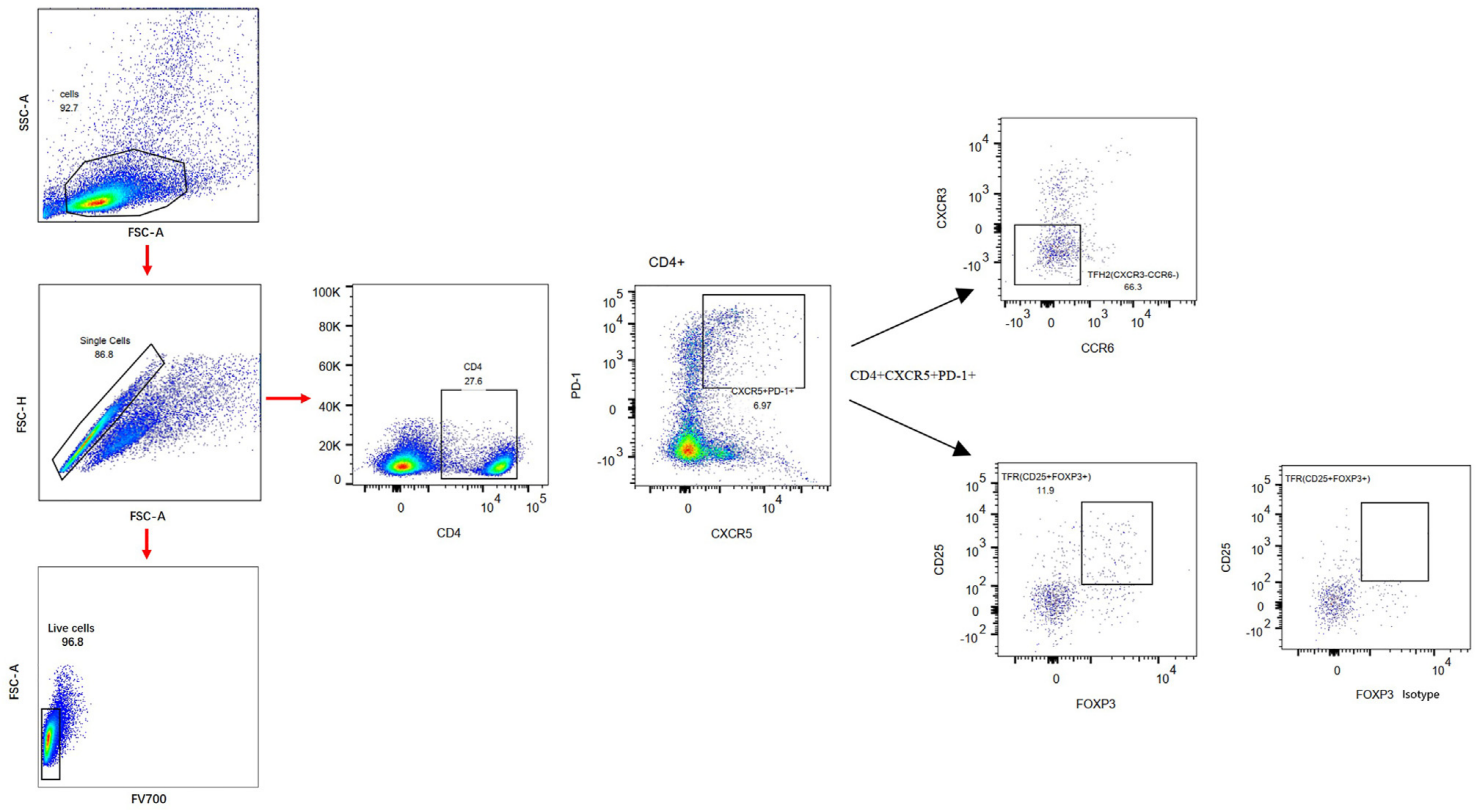
**Gating strategy of peripheral Tfh2 and Tfr cells.** CXCR3^−^CCR6^−^CD4^+^CXCR5^+^PD-1^+^ were defined as Tfh2 cells, CD25^+^Foxp3^+^CD4^+^CXCR5^+^PD-1^+^ were defined as Tfr cells.

The baseline characteristics of the AR patient cohort and control subjects are detailed in Table 1. Our data revealed a statistically significant decrease in serum Apo-AI protein concentrations in AR patients compared to healthy controls (*P* < 0.001), as illustrated in Fig. 1A. Notably, the frequencies of circulating Tfh2 and Tfr cells were also significantly distinct between AR patients and controls (*P* < 0.001), as shown in Fig. 1B–C. The gating strategy for Tfh2 and Tfr was presented in Fig. 2. The serum Apo-AI protein concentrations in AR patients displayed a negative correlation with the Tfh2 cell frequency, as well as serum IL-4 and IL-21 protein concentrations, and the total nasal symptom score (TNSS) in AR patients (Fig. 1D–F-H). Conversely, a positive correlation was observed between serum Apo-AI protein and the frequency of Tfr cell in AR patients (Fig. 1E).

**Table 1:** Demographic characteristic of AR patients and healthy controls

Groups	AR	Control
Number	30	25
Sex (Male, n, %)	18 (60%)	12 (48%)
Age (years)	26.7 ± 6.8	26.1 ± 5.1
Duration of symptoms, (years)	2.4 ± 1.6	–
Serum sIgE level to Der p (IU/mL)	21.7 (5.1–513.8)	–
Serum sIgE level to Der f (IU/mL)	26.9(1.4–296.4)	–

### Apo-AI mediated modulation of Tfh2 and Tfr cell function

**Fig. 3:**
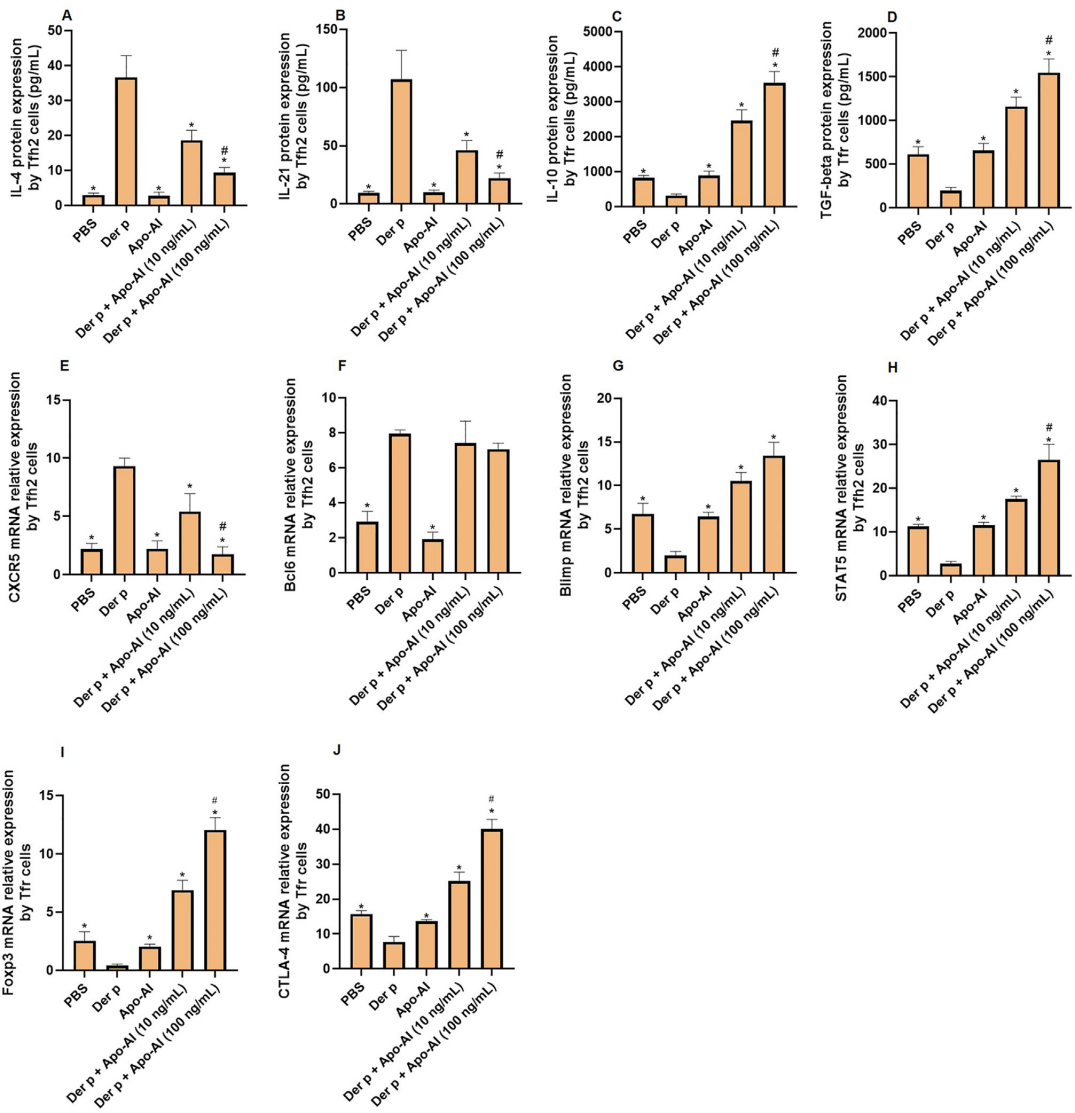
**The regulation of Tfh2 and Tfr cells by Apo-AI.** A,B. ELISA detected the expression of IL-4 and IL-21 protein by Tfh2 cells regulated by Apo-AI (n = 5). C,D. ELISA detected the expression of IL-10 and TGF-beta protein by Tfr cells regulated by Apo-AI (n = 5). E-H. PCR detected the mRNA expression of CXCR5, Bcl6, Blimp and STAT5 by Tfh2 cells regulated by Apo-AI (n = 5). I,J. PCR detected the mRNA expression of Foxp3 and CTLA-4 by Tfr cells regulated by Apo-AI (n = 5). ∗, Compared with Der p group, *P* < 0.05. ^#^, Compared with Der p + Apo-AI (100 ng/mL) group, *P* < 0.05.

To elucidate the direct effect of Apo-AI on Tfh2 and Tfr subsets, we subjected HDM-primed Tfh2 and Tfr cells to a dose-escalation regimen of Apo-AI. Our findings revealed a marked suppression of IL-4 and IL-21 protein expression by Tfh2 cells (Fig. 3A and B), concurrently fostering elevated levels of IL-10 and TGF-β by Tfr cells (Fig. 3C and D). Moreover, the CXCR5 mRNA expression from Tfh2 were downregulated by Apo-AI and the Blimp1 and STAT5 mRNA expression from Tfh2 were upregulated by Apo-AI (Fig. 3E–G,H). The Foxp3 and CTLA-4 mRNA expression from Tfr were upregulated by Apo-AI (Fig. 3I and J). The Bcl6 mRNA expression from Tfh2 were not affected by Apo-AI (Fig. 3F).

### Regulation of Tfh2/Tfr and B cell interactions mediated by Apo-AI

**Fig. 4:**
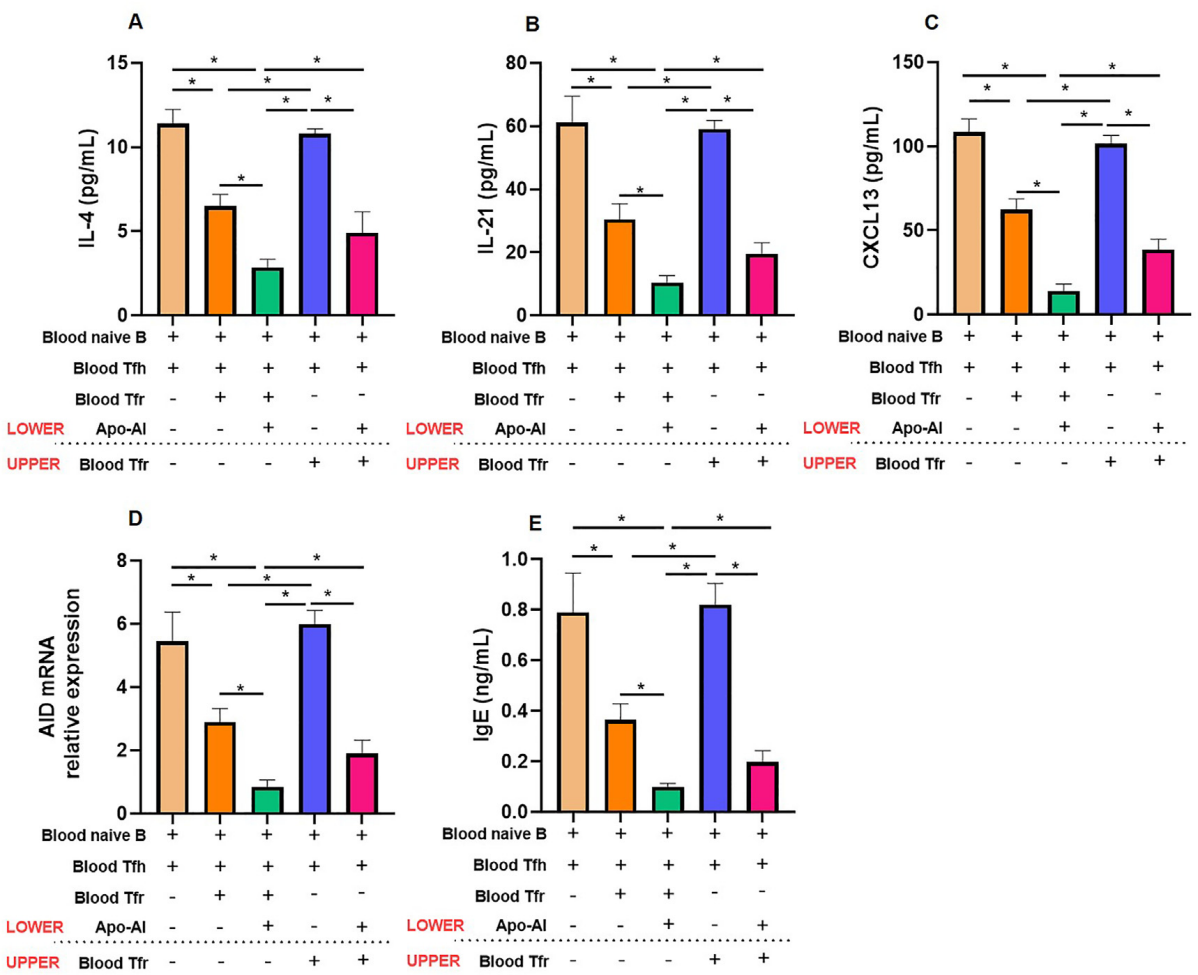
**Inhibitory effect of Apo-AI in Tfr–Tfh2–B-cell coculture system.** Blood Tfh2 and naive B cells from healthy controls were cultured in the lower chamber with or without the presence of Apo-AI, Tfr cells from healthy controls were added in either the upper or lower chamber of Transwell culture system. Cytokine levels (IL-4, IL-21, CXCL13) and IgE were detected by ELISA at day 12 (n = 5). The AID mRNA relative expression was detected by PCR at day 12 (n = 5). ∗, Compared between groups, *P* < 0.05.

To investigate the modulatory role of Apo-AI in the interactions between Tfh2/Tfr and B cells, we employed a well-established Tfr–Tfh2–B-cell coculture system. Our results demonstrate that the addition of Apo-AI in this coculture significantly attenuated the production of key Tfh2 effector molecules, namely IL-4, and IL-21, in the culture supernatants (Fig. 4A and B). Furthermore, Apo-AI downregulated the expression of CXCL13 and activation-induced cytidine deaminase (AID), a pivotal enzyme in B cell activation and antibody class switching (Fig. 4C and D). As expected, the IgE levels were also downregulated by Apo-AI (Fig. 4E).

Crucially, we uncovered that direct cell-cell contact was indispensable for the suppressive function of Tfr cells. When Tfr cells were physically separated from Tfh2 and B cells in a transwell system, their ability to inhibit Tfh2-mediated antibody production by naive B cells was virtually abolished. However, the addition of Apo-AI exerts significant inhibitory effect on Tfh2 and B cells independent of Tfr cells.

### Apo-AI significantly restored the suppressive function of AR-derived Tfr cells

**Fig. 5:**
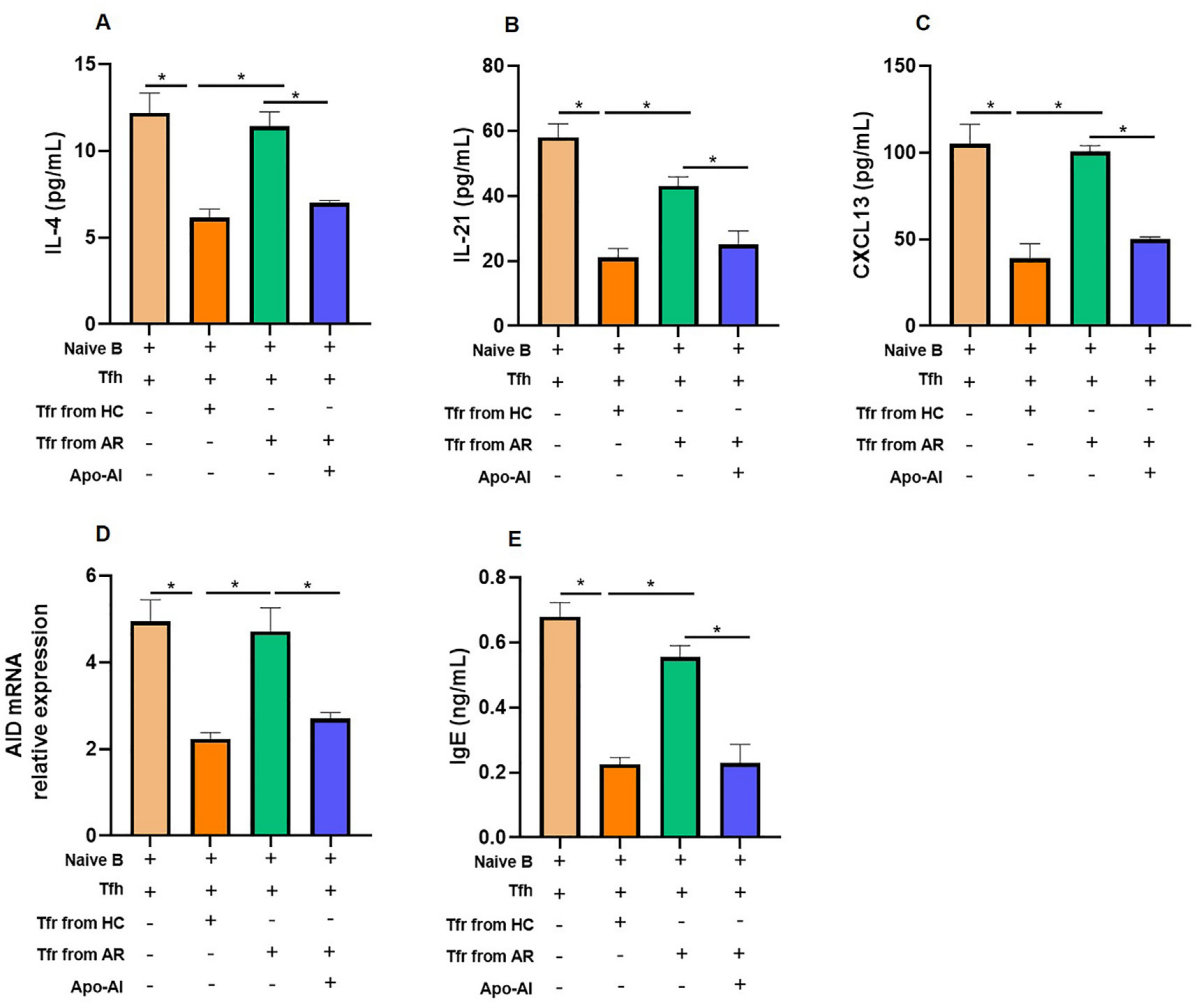
**Apo-AI reverse impaired function of peripheral Tfr cells.** Blood Tfh2 and naive B cells from healthy controls were cocultured with allogeneic Tfr cells from HCs or AR with or without the presence of Apo-AI. A-C, E. Cytokine levels (IL-4, IL-21, CXCL13) and IgE were detected by ELISA at day 12 (n = 5). D. The AID mRNA relative expression was detected by PCR at day 12 (n = 5). ∗, Compared between groups, *P* < 0.05.

Tfr cells isolated from AR patients exhibited a profound impairment in suppressing Tfh2-induced IL-4, IL-21, CXCL13 expression, AID mRNA levels and IgE production by naive B cells, in contrast to Tfr cells derived from healthy controls that effectively mediated this suppression (Fig. 5A–E). Remarkably, the addition of Apo-AI significantly restored the suppressive function of AR-derived Tfr cells (Fig. 5A–E).

### Apo-AI modulates Tfh2/Tfr/B cell function via ICOSL signaling pathway

**Fig. 6:**
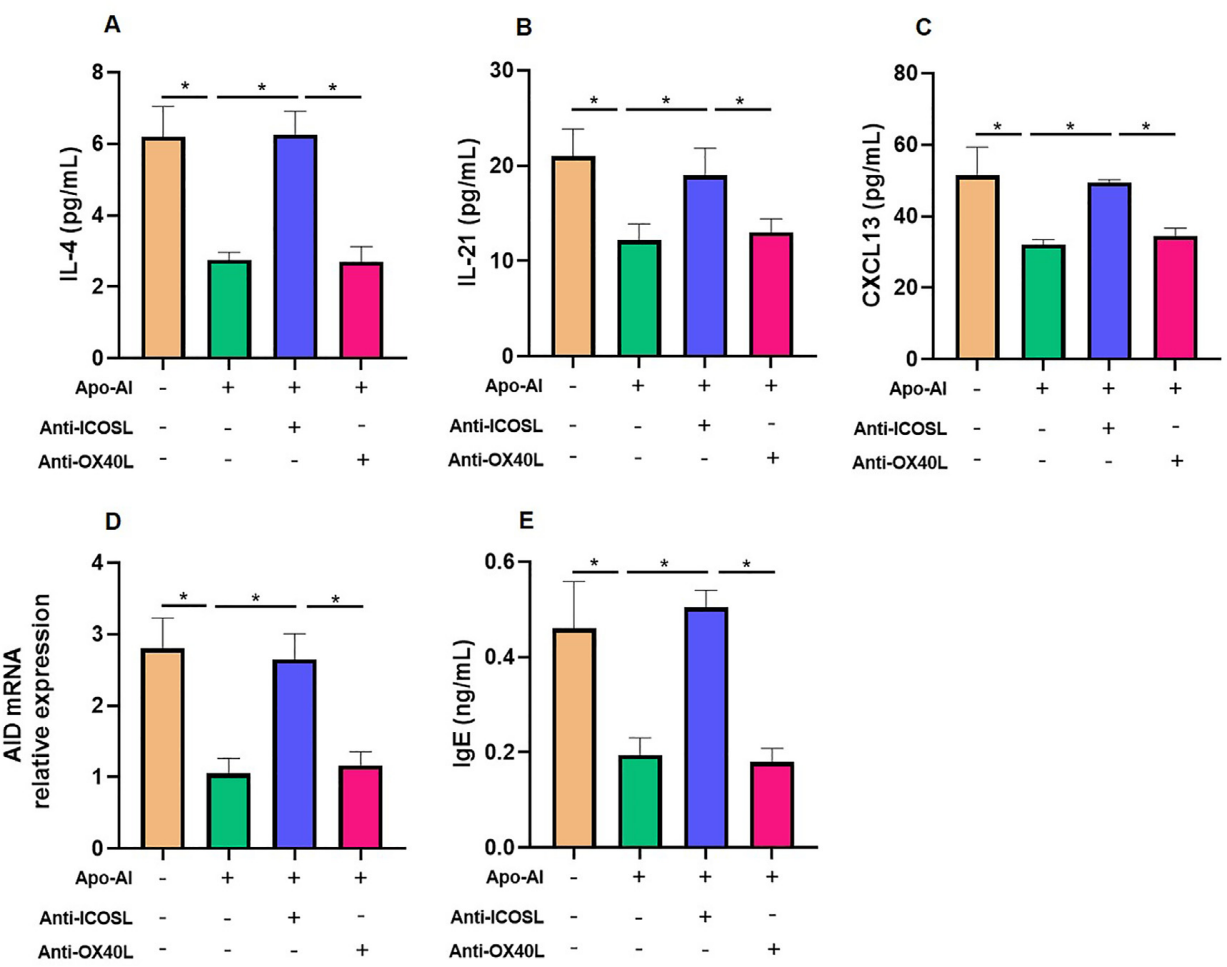
**Apo-AI inhibited function of Tfh2 and naive B cells through ICOSL.** Blood Tfh2, naive B and Tfr cells from healthy controls were cocultured under different conditions. A-C, E. Cytokine levels (IL-4, IL-21, CXCL13) and IgE were detected by ELISA at day 12 (n = 5). D. The AID mRNA relative expression was detected by PCR at day 12 (n = 5). ∗, Compared between groups, *P* < 0.05.

The intricate interplay between Tfh2 and B cells is orchestrated by costimulatory interactions, notably including OX40/OX40L and ICOS/ICOSL pathways, which facilitate mutual assistance during immune responses. Our findings reveal that anti-ICOSL treatments lead to a pronounced enhancement of Tfh2 cell function and the production of IgE in the presence of circulating Tfr cells. This suggests that ICOS/ICOSL signaling is a key pathway for Apo-AI-modulated Tfh2/Tfr/B cell function, whereas the blockade of OX40/OX40L pathways had no effect on the role of Apo-AI. (Fig. 6A–E).

### Apo-AI suppresses Tfh2 and augments Tfr response in an allergic mouse model

**Fig. 7:**
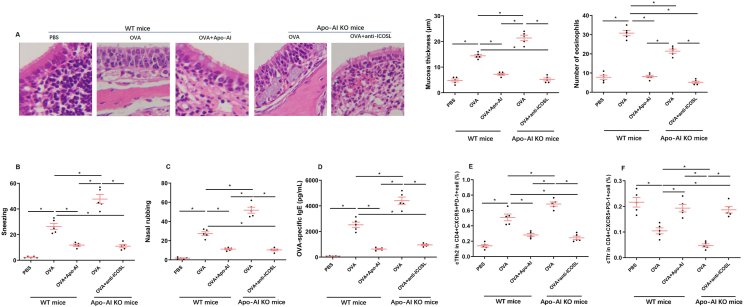
**Apo-AI promoted peripheral Tfr proliferation and inhibited peripheral Tfh2 proliferation in mice model.** A. HE staining of nasal section in different groups. B,C. The nasal symptoms of mice in different groups. D. The serum OVA-specific IgE levels in different groups. E-F. The frequencies of Tfh2 and Tfr in different groups ∗, Compared with groups, *P* < 0.05.

**Fig. S1:**
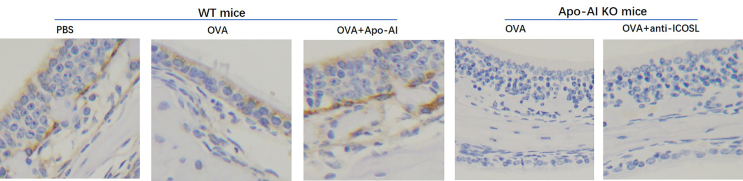
The expression of Apo-AI detected by immunochemistry among various groups

**Fig. S2:**
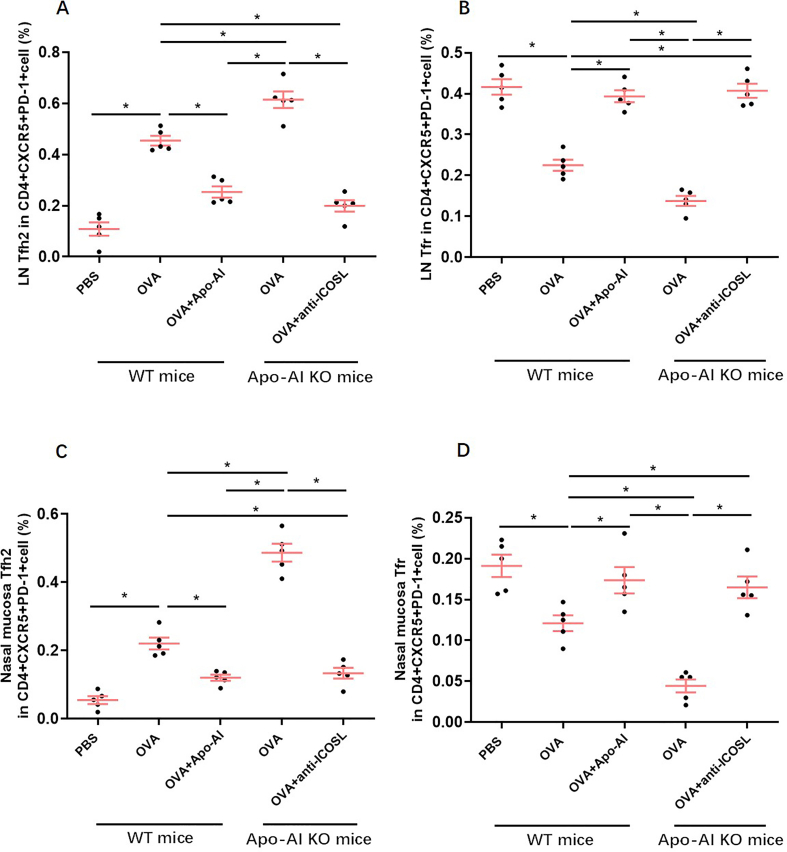
A,B. The frequencies of Tfh2 and Tfr in cervical lymph nodes (LN) among different groups. C,D. The frequencies of Tfh2 and Tfr in nasal mucosa among different groups. ∗, Compared with groups, P < 0.05

We first confirmed the Apo-AI expression in nasal mucosa by immunochemistry and found that the expression of Apo-AI was higher in PBS and OVA + Apo-AI groups compared with OVA group, while Apo-AI expression were not found in Apo-AI KO mice (Supplement Fig. 1). In Apo-AI knockout (Apo-AI^−/−^) mice, a marked increase was observed in eosinophil counts, epithelial thickness, and the numbers of nasal rubbing and sneezing behaviors, accompanied by elevated OVA-specific IgE concentration compared to OVA-sensitized wild-type mice. Conversely, treatment with anti-ICOSL significantly attenuated these allergic manifestations in Apo-AI^−/−^ mice (Fig. 7A–D). As expected, Apo-AI treated OVA-sensitized wild-type mice presented with decreased proportion of Tfh2 cells and increased proportion of Tfr cells in peripheral blood, nasal mucosa and cervical lymph nodes, while Apo-AI knock out mice presented with opposite trend (Fig. 7E and F; Supplement Fig. 2). Notably, anti-ICOSL treatment in Apo-AI^−/−^ mice resulted in a pronounced elevation proportion of Tfr cells and decreased proportion of Tfh2 cells (Fig. 7E and F).

## Discussion

Prior investigations elucidate enhanced Tfh2 cell activity in IgE overproduction of AR patients, whereas Tfr cells exert a suppressive role on Tfh2-mediated B cell antibody generation.^8^ Nevertheless, the precise mechanistic underpinnings of Tfh2/Tfr and B cell interplay remain elusive.

Apo-AI, a pivotal structural and functional constituent of high-density lipoproteins in blood,^15^ exerts multifaceted anti-inflammatory actions on various cells, including monocytes, macrophages, eosinophils, and type 2 innate lymphoid cells (ILC2).^16^, ^17^, ^18^, ^19^ ApoA1 deficiency in mice has been associated with exacerbated lung inflammation, whereas therapeutic intervention with Apo-AI mimetics has demonstrated efficacy in mitigating airway inflammation in animal models.^20^, ^21^, ^22^ In the present study, we present novel insights into the mechanisms by which Apo-AI modulates the function of Tfh2 cells, inhibiting IgE production, and concurrently enhances the activity of Tfr cells, thereby contributing to a more nuanced understanding of its immunomodulatory properties. In Gaddis' investigation, it was observed that ApoAI selectively modulated the transition from exTreg to Tfh cells, without influencing other cellular entities potentially implicated in atherosclerosis progression.^13^ Our research, aligning with previous findings, has validated the suppressive action of Apo-AI on Tfh2 cells and its stimulatory influence on Tfr cells. Future endeavors will delve deeper into elucidating the function of Apo-AI in the transformation between Tfh2 and Tfr cells. Additionally, Black's work demonstrated that transgenic expression of Apo-AI ameliorated systemic lupus erythematosus (SLE)-induced glomerulonephritis in ApoA-Itg mice by decreasing follicular helper T cells, germinal center B cells, and autoantibody levels.^23^ Notably, ApoA-I deficiency did not exert converse effects on autoimmunity or glomerulonephritis, potentially attributable to compensatory elevations of ApoE within high-density lipoprotein (HDL). The discrepancies between their study and ours may stem from the distinct roles that Apo-AI plays in varying disease contexts.

Yao's study had established a positive relation between the frequency of Tfh cells and serum-specific IgE levels to *Dermatophagoides pteronyssinus* (Der p) in AR patients.^6^ In line with these findings, our data reveal a negative association between serum Apo-AI protein levels and Der p-specific IgE production by T follicular helper 2 (Tfh2) cells, whereas a positive correlation is observed with Tfr cells. Notably, we report for the first time that Apo-AI exerts a regulatory role in the differentiation and function of Tfh2 and Tfr cells, modulating key transcription factors such as Blimp1, STAT5, Foxp3, and CTLA-4.

Furthermore, we employed a Tfr-Tfh2-B cell coculture system, confirming the necessity of cell-cell interactions for Tfr-mediated suppression, consistent with Yao's study.^6^ Notably, the addition of Apo-AI significant restored Tfr cell function, suggesting the existence of additional, non-contact-dependent inhibitory pathways mediated by Apo-AI. Moreover, Apo-AI significantly restored the suppressive function of AR-derived Tfr cells, implying its potential treatment target for AR.

The interplay between Tfh and B cells is orchestrated by costimulatory signals, such as OX40/OX40L and ICOS/ICOSL, which facilitate mutual assistance and activation, further emphasizing the complex regulatory network governing humoral immune responses.^24^^,^^25^ The finding that the ICOS/ICOSL pathway plays a key role in Apo-AI-mediated immune regulation is significant because it identifies a specific mechanism through which Apo-AI can modulate immune responses. Despite the broad and multifaceted roles of ICOS/ICOSL in immune regulation, our study highlights a focused interaction between this pathway and Apo-AI, particularly in the context of Tfh2 and Tfr cell function. The broad role of the ICOS/ICOSL pathway in immune regulation may limit its potential as a therapeutic target. However, ICOS/ICOSL has the potential to be a predictive biomarker for the efficacy of Apo-AI-targeted therapies in the future.

To validate our *in vitro* observations, we established an AR mouse model. Concordantly, Apo-AI-deficient mice exhibited reduced percentages of Tfr and more pronounced allergic inflammation, whereas the inflammation was significantly alleviated in anti-ICOSL-treated Apo-AI-deficient mice, implying the key role of ICOS/ICOSL pathway in Apo-AI mediated Tfh2/Tfr regulation.

In conclusion, our data, for the first time, suggest that Apo-AI alleviates AR by regulating the function of Tfh2 and Tfr through both direct and indirect effects, highlighting its potential as a novel treatment target for AR. Based on these findings, we propose that Apo-AI could be explored for its potential application modes in AR treatment, possibly in conjunction with other therapeutic approaches.

## Abbreviations

Tfr, follicular regulatory T cells; Tfh2, type 2 follicular helper T; Apo-AI, Apolipoprotein AI; ICOSL, inducible costimulator ligand; ICOS, inducible costimulator; IgE, immunoglobulin E; TNSS, total nasal symptom score.

## Ethics approval

All patients provided written informed consent at the time of enrollment, and this study was approved by the institutional review boards of Guangzhou Women and Children's Medical Center.

## Author contribution

Wenlong Liu conceptualized and designed the study, drafted the initial manuscript, and approved the final manuscript as submitted. Xiangqian Qiu, Yinhui Zeng, and Jinyuan Li collected the sample, performed the experiment, data collection and statistics. Qingxiang Zeng and Xi Luo reviewed and revised the manuscript. All authors have read and approved the final version of the manuscript.

## Consent for publication

The manuscript's publishing is approved by all of the authors.

## Availability of data and materials

All data generated or analyzed during this study are included in this published article and its supplementary information files.

## Financial support

This study was supported by grants from the National Natural Science Grant of China (No.82271142), Guangdong Special Support Plan for Top Young Talents (No.0720240257), Guangdong Province Natural Science Grant (2024A1515012386), Science and Technology Program of Guangzhou (No. 2023A03J0909, 2024A03J1095, 2024A03J1246), Scientific Research Capacity Improvement Project of Guangzhou Medical University (02-410-2302151XM), Guangxi Natural Science Foundation (2024GXNSFBA010264).

## Declaration of competing interest

The authors declare that they have no relevant conflicts of interest.
